# The Asylum Attendant of To-Day

**Published:** 1892-08-20

**Authors:** 


### HOSPITAL OPINION.

[Contributions critical, suggestive, controversial, and technical are invited for this column, which is open to everyone who has anything to say that is of practical value and interest.]

### THE ASYLUM ATTENDANT OF TO-DAY.

(*By a Medical Snperintendent.*)

We have a very fair sample of the type of nurse now in our asylum wards, in our correspondent, who has bravely taken up the cudgels in defence of asylum attendants and nurses, and who feels hurt at our notice of the subject in a recent review of Mr. Burdett's book on "Asylums of the World." We regret exceedingly that any misconception of our views should have arisen, and have no hesitation in endorsing emphatically the opinion of our correspondent, "Asylum Nurse," regarding the nature of the work which falls to her lot, and the unmistakable advance in character and education to be found in the service at the present time. Mental nursing requires a particular aptitude of its own, wit and intelligence not inferior to that which is necessary in general hospitals, human sympathy, and the faculty of putting one's self in imagination in the place of the unfortunate. This is a catalogue of attributes and virtues of no small importance. Our correspondent has probably the happy experience of being placed in an asylum, where socially and otherwise a superior class of nurses have been attracted into the service, but it is astonishing how diverse are the characters that may be found in different asylums. There is no question about this, that a very strong prejudice exists in many places against asylums as eligible situations for young men and women of education, intelligence, and good character. Asylums are not altogether free from blame for this state of matters, for in many cases the superintendents are antagonistic to movements in favour of improving the training and status of their staff. Yet such superintendents will complain that they can only get inferior persons for their staff, and even those they cannot keep.

In different asylums we may find attendants and nurses who are as opposite to each other as ladies' maids and superior housemaids on the one hand and field labourers and brick workers on the other. Many are scarcely able to sign their names on a pay-sheet, while others use a pen with the facility of a lady clerk, and express themselves in well-rounded sentences. It is fair to admit, however, that the bad writer is not infrequently the better attendant or nurse, for good writing and education are not everything. We are nevertheless forced to the conclusion that what asylum attendants and nurses are, their committees, superintendents, and superior officers are chiefly responsible for. It is with great satisfaction, therefore, that we see to-day a process of evolution going on, a shaking up of the nursing system of asylums, so to speak, a more intimate study of the character and capabilities of our attendants, and the admission of the fact that they are worth the trouble of special training. Ten years ago this last fact was scouted by a large number of superintendents, who pooh-poohed the idea of even a "Handbook of Instruction for Attendants on the Insane." Yet only a few days ago the Medico-Psychological Association of Great Britain and Ireland adopted this handbook as their own, and resolved unanimously to bring out a fresh edition. This resolution, and the recent movement for the training of attendants in asylums, have been frankly accepted by the men who were so slow to move ten years ago; and this is very much to their credit.

The history of lunacy reform teaches that new ideas of the treatment of the insane often owe their origin to outside minds; and that in asylums there has not seldom been shown an inertia and obstinacy inimical to reform, and obstructive in the highest degree. Of late years, however, the zeal of reform has sprung up within asylums themselves. The work of the wards is made more attractive than it used to be, encouragement is given to attendants and nurses to show their own individual taste and talent for ward furnishing and decoration, and no medical officer who really desires the welfare and care of his patients withholds his sympathy and help from a nurse who is honestly endeavouring to know and understand her true duties. The great point is to make a nurse feel that she is a nurse, and not a spoke in the wheel of mechanical discipline. There are nurses and nurses. Some have no interest in anything above the level of common household work; their minds are eternally grubbing amidst dusters and pails and polishing brushes; they devote the working hours of their lives to the mere accidents of asylum life and work. Such persons never realise that their patients are anything but hopeless lunatics, and of a different species from themselves. On the other hand, there are nurses who are intensely humane and sympathetic, who, whilst loving cleanliness, order, and cheerful surroundings, yet recognise that these details, however important they are in themselves, are still secondary to the actual and intelligent management of the unhappy patients. But even with such nurses, encased as they are in a system of tradition and routine, it is comparatively rare to find an individual study of individual cases. They do not often put a question to themselves regarding the nature of a case, or exhibit any particular curiosity about its symptoms and their causes, or the ultimate fate of the patient. The ignorance of many attendants and nurses of the real character of the patients and their maladies is deplorable. Few would care to sit down and engage in conversation with a patient with a view to getting at the real working of his distorted mind. A lunatic sympathetically understood is often a lunatic half cured. Thousands of insane lives are utterly unknown in their inner workings to the staffs of asylums, and it is only when some attendant or nurse with a natural turn for patient inquiry and a shrewd faculty of observation picks up stray threads and pieces together odd traits of insane character, that something like an actual picture of the insane mind and its ways is mentally realised. Other members of the staff are startled for the moment when they find that a silent and apparently expressionless life may have thoughts and feelings after all.

With all the excellence of present attainments in asylums, with all the perfection of our case books, they are but sieves which collect the larger and more apparent facts and allow the smaller, but often weightier, to escape altogether. It would be interesting to apportion accurately to medical officers and nurses respectively their proper share of the facts gleaned for our case books. How much really has the medical officer gathered himself by his own observation during his all too brief stay in the wards, and how much is obtained at second hand from the nurses? If this be so—and no fair-minded man will deny it—it stands to reason that the nursing faculty of observation should be sharpened, and that nurses should be trained and encouraged to observe and make notes of individual cases. At the present moment we are in a transition state. Unquestionably the material of asylum service is being purged of many of the older impurities, moral, intellectual, and otherwise. There is a movement among the dry bones of the old system, and there is a quickening process arising in asylums which is calculated to produce most excellent results. That this may continue until every insane person shall experience the blessings of the rule which enjoines us all to "do as we would be done by" is an aspiration which cannot but arise in every kindly heart.

